# Post-implantation Deformation of Titanium Rod and Cobalt Chrome Rod in Adolescent Idiopathic Scoliosis

**DOI:** 10.5704/MOJ.1903.002

**Published:** 2019-03

**Authors:** U Sia, BB Tan, YY Teo, CC Wong

**Affiliations:** Department of Orthopaedics, Kementerian Kesihatan Malaysia, Hospital Umum Sarawak, Kuching, Malaysia; * Department of Orthopaedics, Universiti Malaysia Sarawak, Kota Samarahan, Malaysia

**Keywords:** adolescent idiopathic scoliosis, thoracic kyphosis, titanium rod, cobalt chrome rod, rod deformation

## Abstract

**Introduction:** Post-implantation rod deformation is anticipated in scoliosis surgery but the difference in rod deformation between titanium and cobalt chrome rod has not been elucidated. This study aims to compare the difference in rod deformation between two groups.

**Materials and Methods:** Twenty-one adolescent idiopathic scoliosis (AIS) patients were recruited from a single center. The over-contoured concave rods were traced prior to insertion. Post-operative sagittal rod shape was determined from lateral radiographs. Rod deformation was determined using maximal rod deflection and angle of the tangents to rod end points. The differences between pre- and post-operative rod contour were analysed statistically. Rod deformation and thoracic kyphosis between two types of implants were analysed.

**Results:** Both rods exhibited significant change of rod angle and deflection post-operatively. Curvature of the titanium rod and cobalt chrome rod decreased from 60.5° to 37°, and 51° to 28° respectively. Deflection of titanium rod and cobalt chrome rod reduced from 28mm to 23.5mm and 30mm to 17mm respectively. There was no significant difference between titanium and cobalt chrome groups with regard to rod angle (p=0.173) and deflection (p=0.654). Thoracic kyphosis was increased from 20° to 26° in titanium group but a reduction from 25° to 23° was noticed in cobalt chrome group, but these findings were not statistically significant.

**Conclusion:** There was no statistical difference in rod deformation between the two groups. Thus, the use of titanium rod in correction of sagittal profile is not inferior in outcome compared with cobalt chrome but with lower cost.

## Introduction

Adolescent idiopathic scoliosis (AIS) is a three-dimensional deformity involving coronal, sagittal and horizontal planes^1^. Multiple postulations have been suggested to explain the pathogenesis of AIS, however, the true etiology remains unknown^2^. In general, surgical treatment is indicated when a curve is greater than 45° or 50°.

The ultimate aim of corrective surgery in AIS is to prevent further curve progression and to obtain a balanced spine. With the evolution of medical technology and spinal instrumentation, deformity correction has improved tremendously over the past decades^3^. Posterior instrumentation and fusion have been a standard of the surgical treatment for scoliosis^4^.

Despite the great evolution of implant material, the advantage of using cobalt chrome implant over titanium implant in scoliosis surgery has not been clearly elucidated. Since there was inadequate evidence that the CoCr rods performed biomechanically better than the titanium rod in restoration of sagittal profile in AIS, the aims of this study were to determine the degree of rod deformation post-implantation, compare the difference in rod deformation between titanium and cobalt chrome rod, assess the correlation between curve flexibility on rod deformation, and to evaluate the effect of direct vertebral rotation (DVR) on thoracic kyphosis (TK).

## Materials and Methods

This is a prospective case series which includes twenty-one patients with Lenke type 1-4 AIS who underwent posterior corrective surgery during the period June 2013 till May 2015 at a single centre. The study was approved by the Medical Research and Ethics Committee (MREC) Malaysia and was registered with the National Medical Research Register (NMRR-17-2075-37751). In this study, all AIS patients aged 13-25 years at the time of operation, treated with posterior spinal fusion with pedicle screw rod system were recruited. Out of 21 patients with adolescent idiopathic scoliosis, 11 patients underwent corrective surgery using cobalt chrome rod and ten patients were treated with titanium rod. Those with rigid scoliosis, kyphotic deformity, required additional correction techniques such as Smith Peterson Osteotomy or Posterior Substraction Osteotomy and those who did not comply with two-year follow-up were excluded from this study.

All operations were performed by a single surgeon with spinal cord monitoring using somatosensory evoked potentials (SSEP) and motor evoked potentials (MEP). All patients were placed in prone position on two pillows with abdomen free for posterior spinal reconstruction. A standard posterior midline skin incision was employed, followed by sub-periosteal dissection of the paravertebral muscles to gain exposure of the posterior bony elements. Pedicle screws were placed at every level on the concave side of the curve using the funnel technique for thoracic vertebra and the inverted V technique (SUK) for lumbar vertebra. On the convex side, pedicle screws were inserted at second or third vertebra levels, with mandatory screws at the upper and lower instrumented levels as well as the apex of the curve. This was followed by soft tissue ligamentous release and facetectomy throughout the instrumented levels. A pre-contoured rod was engaged into the screw head using persuaders on the concave side.

Global rod derotation was done during this rod-screw engaging process without any compression or distraction. After this process, differential derotation using direct vertebral derotation (DVR) technique was performed gradually from the distal most instrumented level. After locking the concave rod in the corrected position, a rod contoured to the corrected curve was placed on the convex side and was locked *in situ*. The final alignment of the spine was adjusted and corrected with either distraction or compression. Lastly, following instrumentation, corticotomy and posterior fusion using autogenous bone were performed.

The Cidambi technique was adopted to analyse the degree of rod deformation after implantation. The contoured concave rod shapes of 5.5mm diameter cobalt chromium (n=11) and 5.5mm diameter titanium spinal rods (n=10) from patients with thoracic adolescent idiopathic scoliosis were traced prior to insertion. The tracings were then digitised into joint photographic experts group format (JPEG). Post-operative sagittal concave rod shape was determined from lateral 2-dimensional radiographs which was performed within a week postoperatively. This post-operative lateral radiograph was selected at this point because we expect the most deformation occurs during operative procedure when the rod was engaged to the pedicle screws. Tracing of postoperative concave rods were again digitised into JPEG. To standardise the measurement, all tracings were processed using Autodesk Revit Architecture whereby the size of the rod was calibrated to their actual 5.5mm diameter.

**Fig. 1:**
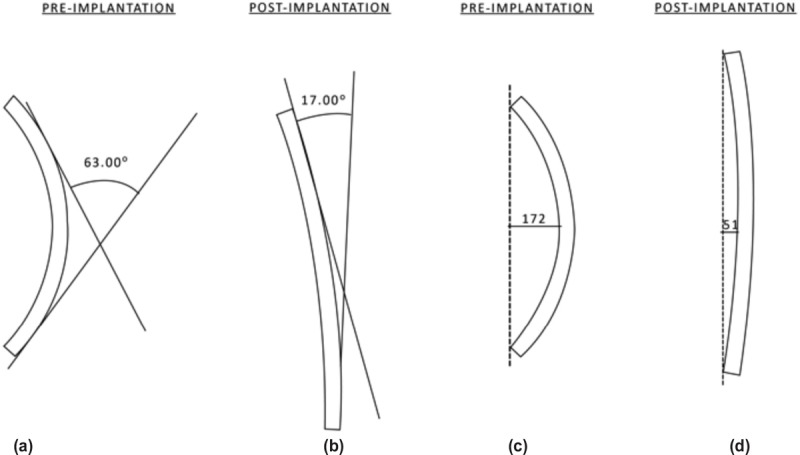
Example of outcome measures. (a) and (b) Show the angle of intersection of tangents to the rod end points. (c) and (d) Show maximal deflection of the rod.

Outcome measures include the maximal rod deflection and angle of the tangents to rod end points (Cobb), method by Cidambi *et al*^5^ (Fig. 1). To establish interobserver reliability, all angle of the tangents to rod end point and rod deflection were measured by three different spine surgeons at different occasions. An average of three measurements in each category was obtained as the final measurement. The differences between pre- and post-operative rod contour were analysed statistically by Wilcoxon signed rank test using a significance level of 0.05. Comparison between two rods was done using Mann-Whitney test. Spearman’s rank-order correlation was used to determine the correlation between fulcrum flexibility and rod deformation.

Baseline radiological studies were assessed to evaluate the severity of scoliotic curve and the magnitude of correction. Pre- and post-operative radiographs were used to obtain Cobb angles and thoracic kyphosis. Correction rate, fulcrum flexibility and fulcrum bending correction index were calculated based on the formula^6^.

## Results

Twenty-one patients with AIS were included in this study. The average age at surgery was 16.8 with the youngest of 12 years old and oldest 25 years old. Average fusion length was 11.3 segments.

Overall, pre-operative Cobb angle was 60.4° while post-operative Cobb angle was corrected to 19.8°. The mean reduction of Cobb angle was 40.6° and a mean correction rate of 68.3% was achieved in this study. A summary of radiological characteristics of titanium and cobalt chrome groups is shown (Table I). There was no significant difference with regard to fulcrum flexibility between two groups (p>0.05). Correction rate was similar for both groups as the difference was not statistically significant.

**Table I:** Radiological characteristics of titanium and cobalt chrome groups

	Titanium group (n=10) Median (IQR)	Cobalt Chrome group (n=11) Median (IQR)	p-Value ^a^
Age at surgery (years)	16 (3)	17 (4)	0.92
Mean Cobb angle of major curve (degree)	59 (15.5)	55 (20)	0.43
Fulcrum flexibility (%)	56 (21)	42 (27)	0.20
Correction rate (%)	70 (14.9)	66.7 (14.5)	0.92

^a^ Mann-Whitney test

Both titanium rod and cobalt chrome rod had reduction in kyphotic shape in all patients, evidenced by reduction in both rod angle and deflection. Rod geometry had changed post-implantation with an average reduction of 21.9° in 21 patients. Curvature of the titanium rod and cobalt chrome rod on concave side decreased from 60.5° to 37° and 51° to 28° respectively. Deflection of titanium rod and cobalt chrome rod reduced from 28mm to 23.5mm and 30mm to 17mm respectively (Table II). Following scoliosis correction, both titanium and cobalt chrome rods were flattened, with a significant decrease in rod angle and deflection.

**Table II:** Comparison of rod deformation before and after implantation for titanium and cobalt chrome groups

	Rod angle Median (IQR)	p-Value ^a^	Deflection Median (IQR)	p-Value ^a^
Pre-operative titanium rod	60.5 (10.4)	0.005	28 (10)	0.005
Post-operative titanium rod	37 (11.6)		23.5 (13)	
Pre-operative cobalt chrome rod	51 (26)	0.003	30 (16)	0.003
Post-operative cobalt chrome rod	28 (22)		17 (19)	

^a^ Wilcoxon signed rank test

There were no significant difference between titanium group and cobalt chrome group with regard to rod angle (P=0.173) and deflection (P=0.654) (Table III). When evaluating thoracic kyphosis (TK), it was slightly increased in the titanium group but a reduction of thoracic kyphosis was noticed in the cobalt chrome group (Table IV). Despite an apparent difference in thoracic kyphosis postoperatively, these findings were not statistically significant.

**Table III:** Comparison of rod deformation between titanium and cobalt chrome group

	Group	Median (IQR)	p-Value ^a^
Rod angle reduction (degree)	Titanium	22.5 (9.9)	0.173
	Titanium Cobalt chrome	17 (7.7)	
Deflection	Titanium	6 (7)	0.654
	Cobalt chrome	8 (5)	

^a^ Mann-Whitney test

**Table IV:** Comparison of thoracic kyphosis between titanium and cobalt-chrome

Variable	Titanium (n=10)	CoCr (n=11)	p-Value ^a^
Pre-operative thoracic kyphosis T2-T12 (degree) Median	20	25	0.56
Post-operative thoracic kyphosis T2-T12 (degree) Median	26	23	0.28
T2-T12 change	+1.5	0	0.61

^a^ Mann-Whitney test

Spearman’s rank-order correlation was used to determine the correlation between flexibility of curve and rod deformation. There was no significant correlation between fulcrum flexibility and rod deformation, measured with both rod angle (p=0.679) and deflection (p=0.758).

## Discussion

Surgical correction remains the mainstay of treatment of AIS. Understanding the natural history of AIS serves as a guide to predict curve progression. Unfortunately, the reported rate of curve progression during skeletal growth varies. Brooks *et al* reported only a 5% incidence of progression of an average of 7° in 474 children with AIS^7^. On the other hand, Soucacos *et al* reported a 14.7% incidence of curve progression of 5° in a larger sample group consisting of 839 children^8^. Nevertheless, substantial number of patients with curves of 50° or more were likely to progress by 1° plus each year and hence needed to be counselled for corrective surgery^9^.

When pedicle screw system gained popularity, the large majority of deformity reconstructions were performed with stainless steel implant because of its strength, *in situ* contouring ability and lack of the notch sensitivity of titanium when bent. A trend switch from using stainless steel implants to titanium alloy for scoliosis surgery started since 2007. Titanium was favourable over stainless steel because of its high biocompatibility, high corrosion resistance, and the ability to perform magnetic resonance imaging without metal artifact^10^. Its disadvantages included decreased stiffness, prone to notching after bending deformation, which made it more liable to fatigue failure.

In recent years, the emergence of cobalt chrome rods has gradually replaced stainless steel rods because it is stiffer and stronger than stainless steel rods, lack of the notch sensitivity of titanium, and MRI compatibility closer to titanium than stainless steel rods. The stiffness of cobalt chrome, estimated by Young’s modulus, is approximately five times stiffer than titanium alloy. This advantage makes cobalt chrome a better implant in deformity correction surgery. Miller *et al* conducted a retrospective comparison of the degree of deformity correction between titanium and cobalt chrome rod in 87 AIS. A favorable result supporting the use of cobalt chrome rods was demonstrated as coronal correction was significantly improved compared to titanium rod using correction rate as outcome measure^11^. Besides the difference of implant materials, constructs with larger rod diameter resulted in stiffer fusion masses and improvement of the magnitude of deformity correction in the axial and sagittal planes^12,13^.

Rod deformation imposes a challenge in our clinical practice because hypokyphosis is a common drawback for scoliosis correction. When it comes to restoration of sagittal profile in scoliosis surgery, many authors and surgeons believe the implant determines the sagittal outcome. Rod contouring or over contouring seems to be an important step to maintain the ideal sagittal profile. However, this maneuver is not always promising as the rod usually deforms and flattens after implantation. To date, virtually no study has compared the rod deformation between titanium and cobalt chrome rod following corrective surgery for AIS.

In this series, there was significant difference between the pre- and post-implantation rod contour after scoliosis surgery, exhibited by both titanium and cobalt chrome rods. Similarly, this finding has long been proven in previous literature^5,14,15^. Debates regarding the superiority of different rod materials in scoliosis surgery persist over the past decade. While many studies suggested that stiffer spinal rod materials such as stainless steel and cobalt chrome rods may exhibit lesser implant rod deformation and deliver higher corrective forces than titanium rods^12,16^, our statistical analysis revealed a negative association between stiffer rod and reduced magnitude of rod deformation. There was similar magnitude of deformation in both titanium and cobalt chrome rod despite the difference in stiffness of both implants.

A mean reduction of 21.9° in rod angle was observed and this finding was comparable with previous studies. Cidambi *et al* reported a reduction in angle of 21° (using 5.5mm ultrahigh-strength steel spinal rods)^5^ while another studies reported a reduction in rod angle of 15.8° (using 6mm titanium rod)^14,15^. In summary, there was no statistical significant difference found between titanium and cobalt chrome rod in term of post-implantation rod deformation based on two outcome parameters. A study by Okada *et al* in their attempt to compare radiological and clinical outcome between stainless steel and titanium instruments in AIS, did not find statistical significant difference at the minimum 2-year follow-up. The stainless steel with a higher stiffness than titanium, exhibited a larger correction loss in coronal plane (4.4 ± 5.2° versus 2.3 ± 5.5°) even though the difference was not statistically significant^17^.

Our hypothesis of the correlation between fulcrum flexibility and rod deformation is surprisingly unsupported. Statistical analysis revealed that there is no significant correlation between fulcrum flexibility and rod angle reduction (p>0.05), and deflection (p>0.05). These results may not reflect the actual phenomenon because of the limitations of this study i.e. under power of sample size and sample not being randomised.

In this series, all our patients had direct vertebral derotation manoeuvre performed during corrective surgery. Pertaining to the myth that vertebral derotation worsens the sagittal profile in scoliosis, our result once again demonstrated similar finding obtained in previous studies^18,19^. Our study showed that application of direct vertebral derotation manoeuvre did not worsen the sagittal profile in adolescent idiopathic scoliosis. There was slight increase of thoracic kyphosis in titanium group (20° to 26°) but slight decrease of thoracic kyphosis in cobalt-chrome group (25° to 23°). However, no statistical difference was demonstrated between these two groups. Miller *et al* demonstrated similar conclusion that the choice of metal alloys between titanium and cobalt chrome did not affect sagittal balance^11^, in contradiction to our result that there was a trend towards better restoration of thoracic kyphosis with titanium.

Comparing with cobalt chrome rod, we postulate that the probable factors contributing to the improvement of thoracic kyphosis with titanium rod are the lower Young’s modulus and the effect of gravity when patients stand in erect position.

Based on our findings, concern of loss of intra-operative rod contouring using titanium implant can be ignored. Kyphosis correction in AIS was not affected by implant material used in this series. In our region, cobalt chrome rod is double the price of titanium rod. In view that the clinical outcome of both implants is similar, titanium rod is more cost effective in the treatment of adolescent idiopathic scoliosis.

The present study was limited by its small sample size and recruited at a single center. In view of the low prevalence of adolescent idiopathic scoliosis, recruitment of similar curve and flexibility is therefore difficult.

## Conclusion

To conclude, this case series compared rod deformation between titanium rod and cobalt chrome rod during corrective surgery in adolescent idiopathic scoliosis. There was no statistical difference in rod deformation between the two groups. Thus, the use of titanium rod in correction of sagittal profile is not inferior in outcome compared with cobalt chrome but is associated with lower cost.
